# Editorial: The Psychology of Suicide: From Research Understandings to Intervention and Treatment

**DOI:** 10.3389/fpsyt.2019.00214

**Published:** 2019-04-04

**Authors:** Yossi Levi-Belz, Yari Gvion, Alan Apter

**Affiliations:** ^1^Department of Behavioral Sciences, Ruppin Academic Center, Hadera, Israel; ^2^The Lior Zfaty Center for Suicide and Mental Pain Studies, Ruppin Academic Center, Emek Hefer, Israel; ^3^Department of Child Clinical Psychology, Bar-Ilan University, Ramat-Gan, Israel; ^4^Feinberg Child Study Center, Schneider Children’s Medical Center of Israel, Petach Tikvah, Israel

**Keywords:** suicide, risk factors, intervention, psychology, treatment

It goes without saying that suicide is a major health problem and a leading cause of death worldwide (1, 2). Recent reports inform that around a million people die by suicide annually, representing an annual global age-standardized suicide rate of 11.4 per 100,000 populations (15.0 for males and 8.0 for females). Considering a time perspective from 2000 through 2016, the age-adjusted suicide rate has grown by 30% (1).

These rates are only the tip of an iceberg. For every suicide, there are many more who attempt suicide every year. A cautious estimate suggests that more than 20 million people engage in suicidal behavior annually. Moreover, it is estimated that in the future, the suicide rates are expected to rise, given the WHO’s declaration that suicide rates will pass the 1 million mark in the next 15 years (2).

Behind each suicide and attempt is a long-term struggle of these individuals as well as experiences of trauma and distress among their relatives and friends. Together, it is evident that suicide prevention comprises a global priority. As clinicians and researchers, we must make every effort to enhance suicide prevention in order to improve our identification, intervention, and, subsequently, prevention of suicide and suicidal behavior. First and foremost, our mission is to improve our knowledge of mechanisms, factors, and facilitators of suicidality from interdisciplinary perspectives.

Suicide is a highly complex and multifaceted phenomenon, with many contributing and facilitating variables. It may be determined by the interaction between various factors, such as neurobiology, personal and family history, stressful events, and sociocultural environment (3). Given its being one of the most severe human behaviors, a distinct focus would be to identify the underlying psychological processes that may lead to suicidal ideation and behavior.

In the last century, we have recognized the contributions of psychological factors (both individual and social) to suicide and suicide risk. A number of models have been proposed, with most emphasizing the interaction between predisposing and precipitating factors (4, 5).

The key factor leading to suicide is unbearable mental pain (6). Several studies have emphasized the importance of psychache as the primary facilitator of suicide ideation and behavior (7, 8). Suicide can be seen as a behavior motivated by the desire to escape from unbearable psychological pain (9, 10). Other psychological factors like personality traits, emotional characteristics, and dysregulation also seem to play a role, with emerging importance to decision-making deficit among suicidal individuals (11).

Interpersonal factors also play an essential role in suicides. Emile Durkheim’s (12) seminal work established the foundations of our understanding that suicide is also a social behavior having some cultural characteristics. Joiner’s interpersonal theory of suicide (13) highlights two major interpersonal structures—perceived burdensomeness and thwarted belongingness—as critical features that may lead to suicidal ideation and eventually to suicide.

Approximately 45% of individuals who die by suicide consult a primary care physician within 1 month of death, without declaring their suicide desires and ideation (14). This finding highlights the fact that communication difficulties comprise a major focus of our understanding of suicidal behavior. In the Israeli MSSA (Medically Serious Suicide Attempters) project, Levi-Belz and colleagues showed that poor self-disclosure, together with several related factors, may facilitate more lethal suicide behavior (15–18).

These examples of studies are representative of numerous endeavors to deepen our understanding of the psychology of suicide phenomenon. In order to continue this course of action and thought, we dedicate a special issue of *Frontiers in Psychiatry* to the effort to explore various approaches to the psychology of suicidal behavior. The purpose of the current issue is to shed light on in-depth knowledge and empirical data regarding models, theories, and specific dimensions and variables that may help us increase the psychological understanding of suicidal phenomena as well as non-suicidal self-injury (NSSI).

Five stimulating reviews are presented in this issue. Gvion and Levi-Belz examined specific risk factors for serious suicide attempts (SSAs). SSAs are epidemiologically very similar to those who died by suicide and thus may serve as valid proxies for studying suicides. The authors conclude that the interaction of mental pain, interpersonal factors, and impaired decision making is crucial for suicide risk assessment and research. Szücs et al. focus on personality and suicidal behavior in old age in their systematic review. Their review of 31 scientific papers emphasized that maladaptive personality manifests in milder, subthreshold, and more heterogeneous forms in late-life vs. early-life suicide. Moreover, the inability to adapt to changes occurring in late life may explain the relationship between suicide in old age and higher conscientiousness. Obsessive–compulsive and avoidant personality traits were particularly associated with elderly suicide.

Cipriano et al. conducted an up-to-date systematic review on NSSI, focusing on epidemiological, etiologic, and diagnostic criteria. NSSI was found to be most common among adolescents and young adults. Borderline personality disorder and eating disorders are reported as comorbid antecedents for NSSI. Prevalence rates are 7.5–46.5% for adolescents, 38.9% for university students, and 4–23% for adults. In a mini-review article, Geraldo da Silva et al. group the main cognitive difficulties among individuals who attempt suicide. These include attentional bias, impulsivity, and problem-solving and decision-making deficits. They suggest that in addition to anxiety and depressive symptoms, cognitive deficits in psychiatric patients comprise important therapeutic goals. Finally, in Conti et al.’s review, the authors systematically review the relations between binge eating disorder (BED) and suicidal ideation and suicide attempts. They found that BED is significantly associated with a marked increase in suicidal behaviors and ideation.

Three papers focus on identifying risk factors in childhood. Schmidt et al. used structural equation modeling to test theory-driven models in clinical high risk (CHR) for psychosis. CHR patients were particularly prone to suicidality if adversity/trauma was followed by the development of depressiveness. In addition, adversity/trauma led to suicidality through an increased risk for psychosis as indicated by cognitive symptoms.

Bar-Zomer and Brunstein-Klomek examined associations between sibling bullying, attachment to mother and father, depression, and suicidal ideation among students. Bullying among siblings was associated with school bullying, depression, and suicide ideation. A secure attachment to one’s father moderated the association between sibling bullying and depression/suicide ideation.

In a third article, Falgares et al. assessed the role of self-criticism and dependency as potential mediators of the link between different types of childhood maltreatment and suicide among university students. Lack of care and psychological abuse were significantly associated with suicide risk, and this association was partially mediated by the maladaptive personality dimension of self-criticism.

Several studies examined risk factors of suicidal behavior in specific populations. Stein et al. observed a sequential model in their longitudinal study among former prisoners of war (ex-POWs) in Israel. They found that PTSD symptoms facilitated experiencing loneliness, and these worked in tandem to implicate suicidal ideation, even years following their captivity. They conclude that both PTSD symptoms and loneliness are important factors in ex-POWs’ long-term suicidal ideation and risk. Mérida-López et al. examined the role of emotional intelligence (EI) as a protective factor of suicide ideation and behavior among students and the general population. They found EI to be related to suicide risk, with psychological distress as a mediator. They concluded that the underlying process by which self-reported EI may act as a protective factor against suicidal ideation and behaviors is through the reduction of distress among those with high EI.

Balazs et al. examined quality of life (QoL) as a factor that may serve as a link between psychopathology and suicide risk among a clinical population of adolescents. QoL significantly mediated the relationships between emotional difficulties and peer problems, as both were associated with lower QoL, which, in turn, was related to higher suicidal risk. Hofstra et al. focus on time trends of suicide among the Dutch population. They observed 33,224 suicide events that occurred from 1995 to 2015. Results indicated that suicide incidence peaked at springtime and on Christmas, which highlights the importance of accessibility of health care services during these high-risk moments.

Several studies in this issue suggested other important risk factors. Tavakoli et al. examined the association between attentional control and suicidal behavior among a cohort of inpatient adolescents presenting acute suicidal behavior compared to healthy controls. A passively presented auditory optimal paradigm was used. The extent of processing these “to-be-ignored” auditory stimuli was measured by recording event-related potentials (ERPs), which are thought to reflect processes linked with capturing attention. The study found a relatively low threshold for the triggering of the involuntary switch of attention among suicidal patients, a factor that may play a role in their reported distractibility. Thompson and Chen Ong investigated the association of suicidal behavior with neurological and behavioral markers, measuring attentional bias and inhibition in two Stroop tasks, as well as recorded activity in frontal areas by EEG (Electroencephalogram) during each task. High-risk participants showed slower response times in the color Stroop (as well as to the word “suicide”) and reduced accuracy in incongruent trials, but faster response times in the emotional Stroop task (with reduced activity in leftward frontal areas). Results confirmed that suicide attempters have deficits in attentional control that may be related to particular conditions of frontal asymmetry. In another important study, Hadlaczky et al. examined the relationship between loss aversion and suicidal behavior among adolescents recruited in 30 schools in seven European countries. Loss aversion predicted attempted suicide in both cross-sectional and 4-month prospective analyses after controlling for depression, anxiety, stress, and gender. Interestingly, loss aversion did not predict suicide ideation in this group.

Several innovative papers examine the essential topic of therapeutic interventions for suicidal patients, with a particular focus on reducing suicide rates. First, Iyengar et al. conducted a systematic review of randomized controlled trials, reporting therapeutic interventions as being effective in reducing self-harm, including suicide attempts (SA). Also reported was a reduction of suicidal ideation and depressive symptoms following therapeutic interventions. While most of the studies were unable to determine the efficacy of therapeutic interventions for both primary and secondary outcomes, individual self-driven and socially driven processes seemed to display the greatest prospect of reducing suicide attempts.

Another contribution on prevention is that of Pickering et al., who studied an intervention program in which students underwent a yearlong training as peer leaders, and 3,730 9th−12th graders completed baseline surveys assessing friendships and adults at school as well as recording suicidal thoughts and behaviors. In general, training more peer leaders increased school-wide exposure for all modalities. Exposure was higher for students closer to peer leaders in the friendship network and for students who named more trusted adults. Relatedly, Barzilay et al. validated the Therapist Response Questionnaire-Suicide Form (TRQ-SF) in a general outpatient clinic setting in a cohort of adult psychiatric outpatients and their therapists. TRQ-SF correlated positively with concurrent and predictive evaluations of patient suicidal outcomes, depression severity, and clinicians’ judgment of patient suicide risk. However, the TRQ-SF was not predictive of global symptom severity, thus indicating specifically suicide-related responses. In a seminal work, Brodsky, et al. present the assess, intervene, and monitor for suicide prevention model as a guide for implementation of the Zero Suicide model, a framework to coordinate a multilevel approach for applying evidence-based practices in clinical settings. The paper describes 10 basic steps for clinical management and illustrates how to implement them through a clinical vignette. Finally, Sakashita and Oyama present an integration of psycho-behavioral components associated with suicide, existing guidelines for identifying critical points of intervention, and the preventive strategies framework into a theoretical model for elderly suicide.

Two more papers examine different issues regarding suicide. Moreno-Küstner et al. focus on demographic factors in their analysis of the characteristics of 181,824 calls made to the Málaga Prehospital Emergency Service for suicidal behavior. Of the total calls (*N* = 181,824), 1,728 (0.9%) were made due to suicidal behavior. The mean age was 43.21 ( ± 18) years, and 57.4% were women. Zhao and Shai’s study reveals that the students’ attitudes toward suicidal behavior and the attitude toward suicide-loss survivors played as a mediator between self-efficacy in managing happiness and self-evaluations among college students. Thus, for this population, the attitudes toward suicide may be understood as one of the factors that shape self-evaluation and satisfaction with life.

In conclusion, in this special issue, we seek to advance the knowledge on suicide by identifying particular psychological characteristics that may facilitate targeted prevention, intervention methods, and programs. Improving our understanding of these topics may help clinicians and researchers establish specific prevention strategies and methods that will ultimately help diminish suicide rates around the globe as well as find psychological remedy for all of those struggling with suicide ideation and behavior.

## Author Contributions

We state that 1) all authors have read the paper and approved the data and the conclusions presented therein; 2) each author believes that the paper represents honest work; 3) all authors have contributed to the present paper with equal effort; and 4) no financial support was given for this editorial.

## Conflict of Interest Statement

The authors declare that the research was conducted in the absence of any commercial or financial relationships that could be construed as a potential conflict of interest.
